# Supplementary material for: Transmembrane Polar Relay Drives the Allosteric Regulation for ABCG5/G8 Sterol Transporter
Source: Int J Mol Sci. 2020 Nov 19;21(22):8747. doi: 10.3390/ijms21228747 (PMC7699580; doi:10.3390/ijms21228747)
Supplement: Supplementary file 1 [file ijms-21-08747-s001.pdf]

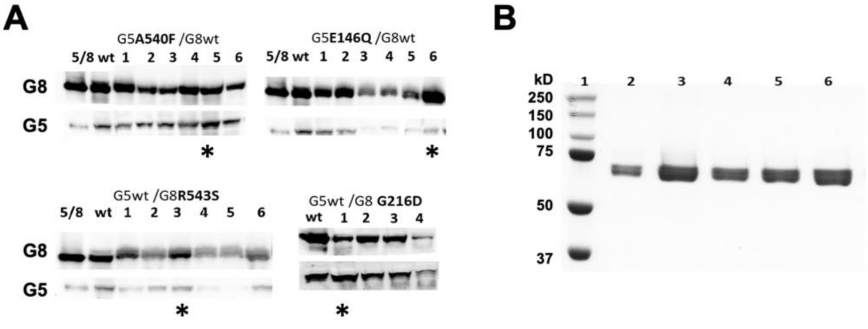

Figure S1. Expression and purification of ABCG5/G8 missense mutants. (**A**) Four or six yeast colonies were selected for expression test. Crude microsomal membranes (either WT or mutants), containing 20–30μg total proteins, were resolved by SDS–PAGE. Protein expression was analyzed by Western blotting using a monoclonal anti-RGS-His antibody to detect ABCG5 and a polyclonal anti-human ABCG8 antibody to detect ABCG8. The clones expressing the highest level for both subunits were selected for protein purification. Selected clones are indicated as asterisks. (**B**) Gel-filtration purified mutants were resolved on a 10% SDS-PAGE gel and stained by Coomassie Blue (shown here in greyscale). Lanes 1: molecular weight marker, 2: G8-G216D, 3: G5-E146Q, 4: G8-R543S, 5: G5-A540F, and 6: WT.

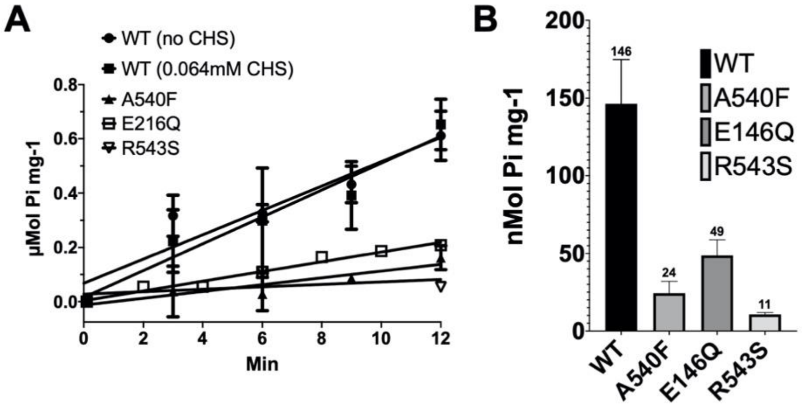

Figure S2. Non-CHS-stimulated ATPase activity of ABCG5/G8. (**A**) The ATP hydrolysis by WT or mutant ABCG5/G8 was measured at 37°C in presence of 5mM ATP and 0.064mM CHS, a condition that resulted in consistent measurement of mutant-mediated ATPase activity and that has no appreciable effect on the WT-mediated ATPase activity. An assay protocol is described in Materials and Methods. The data points are presented as the means ± standard deviations of duplicated or triplicated experiments by using 2–4 independently purified protein preparations, where not visible, the error bars are covered by the plot symbols. A linear regression, plotted from the first 12 minutes, is used to calculate the specific activities. (**B**) Bar graphs show the specific activities of non-CHS-stimulated ATP hydrolysis by WT and mutants. A540F: sterol-binding mutant G5-A540F; E146Q: sitosterolemia mutant G5-E146Q; R543S: sitosterolemia mutant G8-R543S.

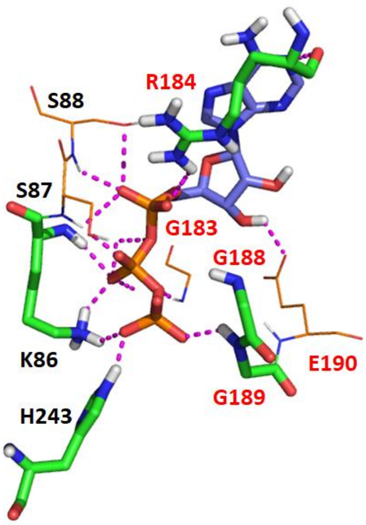

Figure S3. The interaction between ATP and ABCG2 revealed by a representative MD snapshot. ATP is shown as bluish sticks and hotspot residues in greenish sticks ($\Delta G_{lig\text{-}res} \leq -10.0$ kcal/mol). Other residues forming hydrogen bonds (dashed magenta lines) with ATP are shown in lines.

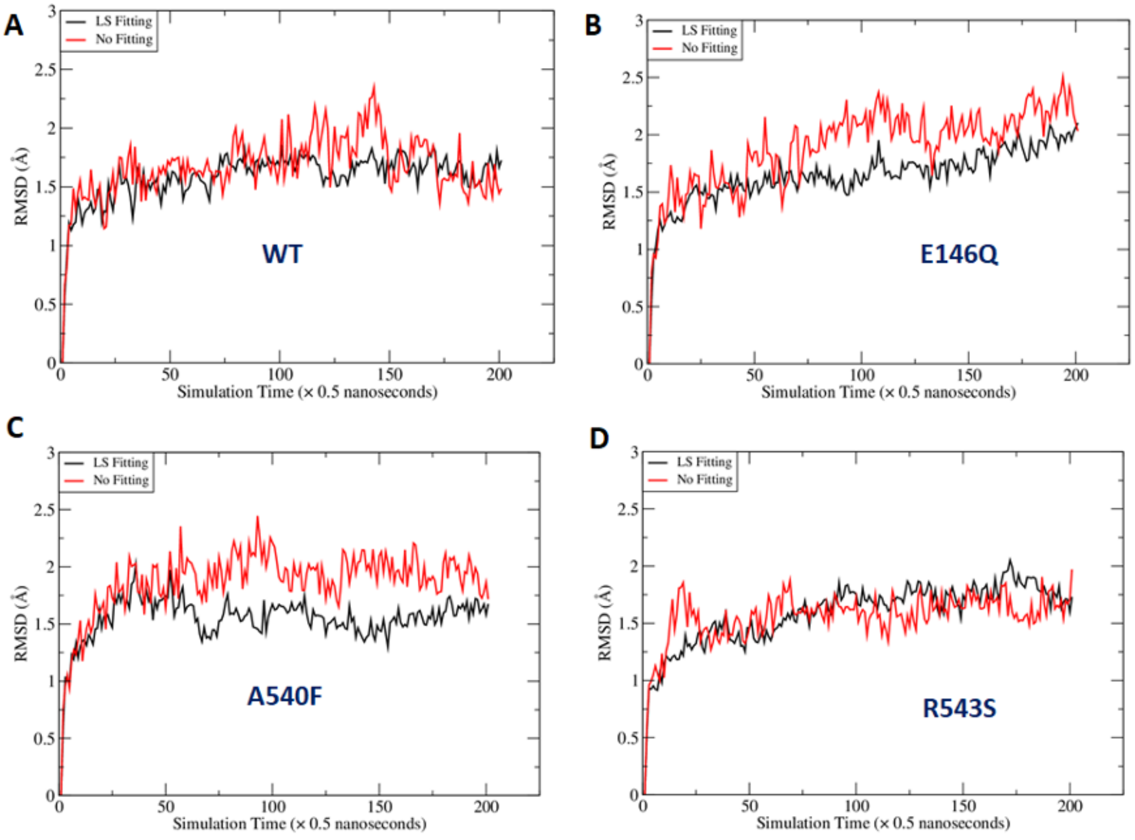

Figure S4. Fluctuation of Root-mean-square deviations (RMSD) along MD simulation time course. RMSD were calculated for the hypothetical residues surrounding ATP under two scenarios. In the first scenario, least-square (LS) fittings were performed for the main chain atoms of the hypothetic residues which including Residues 88–103, 246–251 of G5 and 210–220, 237–245 of G8. In the second scenario, the RMSD were calculated directly for the hypothetical residues without LS Fitting after the MD snapshots were aligned to the crystal structure using the secondary structures of ABCG5/G8. The fluctuations of the first and second scenarios were illustrated using black and red curves. (**A**) wild type; (**B**) E146Q mutant in ABCG5; (**C**) A540F mutant in ABCG5; (**D**) R543S mutant in ABCG8. G5G8: ABCG5/G8; SS: secondary structure.

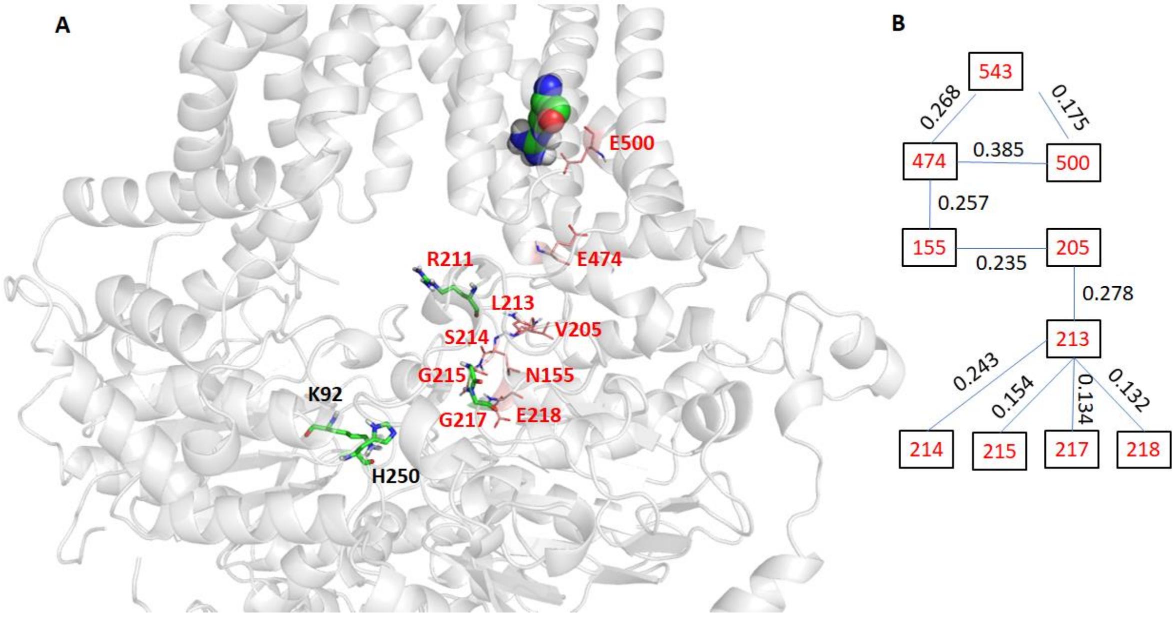

Figure S5. Interaction pathways link Residue R543 of ABCG8 and hotspot residues. The hotspot residues which have ligand-residue interaction energies more potent than -10.0 kcal/mol are shown in sticks, and residues are within the interaction pathways are shown in lines and labeled in red (Panel **A**). The residues within the interaction pathways and the correlation between them are shown in Panel (**B**). A correlation between two residues, which is between 0 and 1, was obtained through correlation analysis. The average correlation for all wild-type ABCG5/G8 pairs is 0.0018 and the minimum and maximum are 0 and 0.52, respectively.

Table S1. List of the hotspot residues for ABCG2 and the corresponding residues in ABCG5/G8. Hotspot residues that have ligand-residue MM-GBSA energies smaller than −7.0 kcal/mol, are shown in red.

Chain A					Chain B				
ABCG2			ABCG5/G8		ABCG2			ABCG5/G8	
ID	Type	$\Delta G_{lig\text{-}res}$	ID	Type	ID	Type	$\Delta G_{lig\text{-}res}$	ID	Type
82	T	-7.8	88	S	183	I	-0.1	210	V
83	G	-6.1	89	G	184	R	-21.7	211	R
84	G	-3.8	90	S	185	G	-1.8	212	G
85	G	-7.6	91	G	186	V	-3.7	213	L
86	K	-58.8	92	K	187	S	-6.1	214	S
87	S	0.0	93	T	188	G	-10.1	215	G
88	S	-1.7	94	T	189	G	-10.6	216	G
89	L	-1.3	95	L	190	E	-7.1	217	E
90	L	-1.4	96	L	191	R	-3.5	218	R
91	D	0.0	97	D	192	K	-2.1	219	R
92	V	-0.2	98	A	193	R	-3.9	220	R
93	L	-0.1	99	M					
94	A	0.0	100	S	210	D	0.0	237	D
95	A	0.0	101	G	211	Q	-0.1	238	E
96	R	-0.3	102	R	212	P	0.0	239	P
97	K	-5.3	103	L	213	T	0.0	240	T
					214	T	-1.3	241	S
239	I	-0.2	246	V	215	G	0.0	242	G
240	F	-0.9	247	L	216	L	-1.3	243	L
241	S	0.0	248	T	217	D	0.0	244	D
242	I	-0.2	249	I	218	S	0.0	245	S
243	H	-12.6	250	H					
244	Q	-0.4	251	Q					
